# Immuno-PET: Design options and clinical proof-of-concept

**DOI:** 10.3389/fmed.2022.1026083

**Published:** 2022-10-14

**Authors:** Alexandre Lugat, Clément Bailly, Michel Chérel, Caroline Rousseau, Françoise Kraeber-Bodéré, Caroline Bodet-Milin, Mickaël Bourgeois

**Affiliations:** ^1^Nantes-Angers Cancer Research Center CRCI2NA, University of Nantes, INSERM UMR1307, CNRS-ERL6075, Nantes, France; ^2^Nuclear Medicine Department, University Hospital, Nantes, France; ^3^Department of Nuclear Medicine, Institut de Cancérologie de l'Ouest (ICO) – Site Gauducheau, Saint-Herblain, France; ^4^ARRONAX Cyclotron, Saint-Herblain, France

**Keywords:** nuclear medicine, immuno-PET, diagnosis, theranostic, monoclonal antibody

## Abstract

Radioimmunoconjugates have been used for over 30 years in nuclear medicine applications. In the last few years, advances in cancer biology knowledge have led to the identification of new molecular targets specific to certain patient subgroups. The use of these targets in targeted therapies approaches has allowed the developments of specifically tailored therapeutics for patients. As consequence of the PET-imaging progresses, nuclear medicine has developed powerful imaging tools, based on monoclonal antibodies, to *in vivo* characterization of these tumor biomarkers. This imaging modality known as immuno-positron emission tomography (immuno-PET) is currently in fastest-growing and its medical value lies in its ability to give a non-invasive method to assess the *in vivo* target expression and distribution and provide key-information on the tumor targeting. Currently, immuno-PET presents promising probes for different nuclear medicine topics as staging/stratification tool, theranostic approaches or predictive/prognostic biomarkers. To develop a radiopharmaceutical drug that can be used in immuno-PET approach, it is necessary to find the best compromise between the isotope choice and the immunologic structure (full monoclonal antibody or derivatives). Through some clinical applications, this paper review aims to discuss the most important aspects of the isotope choice and the usable proteic structure that can be used to meet the clinical needs.

## Introduction

The original idea of personalized and tailored drugs, highly specific to a pharmacological target, appeared at the beginning of the XXth century via the “magic bullet” concept (1) brought by Paul Ehrlich (Nobel laureate in 1908). In oncologic field, this postulate aims to optimize the risk-benefit ratio for the patients care. Yet, it was not until the late 1970's that the discovery of hybridoma technology by Georges Kohler and Cesar Milstein (Nobel laureates in 1984) made it possible to apply this theory in clinical practice. The hybridoma technology allowed biochemists and immunologists to produce monoclonal antibodies (mAbs) which specifically recognize antigens on pathologic cells, thereby providing the proof-of-concept for specific immunotherapeutic approaches to treat cancer (2).

With the concomitant progress in nuclear medicine, the radiolabeled monoclonal antibody (mAb) quickly emerged to specifically target antigens of abnormal cells, most often cancerous. The discovery of overexpressed antigens in many hematologic and solid tumors paved the way for the use of gamma emitters such as iodine-131 (^131^I) radiolabeled mAbs for tumor detection in nuclear medicine practice (3, 4). In parallel, iodine-131 being also a beta minus emitter, the use of ^131^I-mAbs has quickly found its place in therapeutic approaches, mainly in hematologic neoplasms with real efficacy in B-cell lymphoma (5). For almost three decades, mAbs have been labeled with gamma emitters (such ^131^I or ^111^In) in planar or Single Photon Emission Computed Tomography (SPECT) imaging procedures. Despite the reliable and confident clinical information, this modality suffers from several drawbacks including poor sensitivity, poor spatial resolution and complex scatter correction due to the collimator.

While Positron Emission Tomography (PET) was revolutionizing diagnostic applications of nuclear medicine with small organic molecules such as fluorodeoxyglucose (FDG) radiolabeled with fluorine-18 (^18^F), PET approach seemed unusable with long pharmacokinetic half-life mAbs. The availability in the early 2000s (6–8) of longer half-life isotopes such as iodine-124, copper-64 or zirconium-89 allowed the fruitful association between the high specificity of mAbs with the high resolution of PET leading to the Immuno-PET approach. Comparatively to classical SPECT modality, using of immuno-PET imaging combined several technical advantages such as precise scatter correction, exact correction of attenuation, accurate quantitative information, improved spatial resolution with a better delineation of tumors and organs, and, last but not least, higher sensitivity associated with the capacity to perform a true whole-body imaging in a reasonable time. The recent technological evolution in new PET detectors and reconstruction algorithms (9–11) appeared to be also a key factor for the immuno-PET clinical performance in terms of both spatial resolution and signal-to-noise ratio.

Today, the overall performance of the immuno-PET methodology is carried by different biological or imaging parameters such target accessibility or imaging protocol (time between injection and image acquisition, number of acquisitions). The aim of this paper review is more focused on the pharmacological choices to find the best matching between the radionuclide parameters (physical, chemical, logistic) and the mAb used (specificity, affinity, dose, mAb derivative, pharmacokinetic…). Immuno-PET is a fast-growing approach (12) in many cancer pathologies (13). Currently, from an imaging point of view, the management of patients suffering from a tumor pathology is mainly driven on an initial diagnosis made by conventional techniques combining CT/MRI and ^18^F-FDG PET. In this care management, immuno-PET approach finds a particularly effective place to provide some specific information on the tumor phenotype, intra- and inter-tumor heterogeneity and as prognostic/predictive indicator for targeted therapies. It should be noted that in the specific case, where the immuno-PET can be used to select only patients in whom the treatment is likely to provide a benefit, the immuno-PET falls into the scope of companion diagnostic. Some preclinical proof-of-concept will be discussed in mAb derivatives section and a clinical part will illustrate, through some typical examples of results obtained with compounds (mAb and immunological derivatives) allowing antigen targeting in solid tumors, hematological and tumor microenvironment.

## Isotope choice for immuno-PET

One of the immuno-PET key success is based on an appropriate matching between the biological half-life of the immunoprotein and the physical half-life of the isotope to achieve the best contrast in pathologic tissues (14–16). Despite the great variety of radionuclides which are positron emitters, only few of them could be used in immuno-PET. For nuclear medicine applications, the choice of the radionuclide is basically based on three main criteria: physical characteristics, chemical characteristics, and production/logistic. The list of isotopes used (or considered) is summarized in Table 1. Indeed, intact mAbs have a pharmacokinetic half-life of several days with a long circulation time and required a long radionuclide half-life to increase the tumor-to-background ratio such as iodine-124 or zirconium-89. Copper-64 with its intermediate half-life can be used for labeling a large size range of molecules like native mAbs or their smaller derivatives [F(ab')_2_, F(ab), minibody, ScFv, nanobody or affibody structures]. Fluorine-18 and gallium-68 with their shorter half-life may be used to label small molecular weight molecules such as mAb derivates or small synthetic molecules for pretargeting approach (15).

**Table 1:** Usable radionuclides for immuno-PET.

**Radionuclides**	**Half-life**	**Positron emission β^+^ intensity (Mean energy)**	**Main other emission type (energy; intensity)**	**Chemical**	**Binding**	**Production way**
^68^Ga	67.7 min	88.9% (829.5 keV)	γ (1077 keV; 3.22%)	Metallic	Complexation (DOTA, HBEDCC)	Cyclotron → generator
^18^F	109.7 min	96.7% (249.8 keV)	/	Halogen	Covalent (direct/prosthetic)	Cyclotron
^44^Sc	3.97 h	94.3% (632.0 keV)	γ (1157 keV; 99.9%)	Metallic	Complexation (DOTA)	Cyclotron
^64^Cu	12.7 h	17.5% (278.0 keV)	γ (1346 keV; 0.47%) β^−^ (190.7 keV; 38.5%)	Metallic	Complexation (DOTA, NOTA, TEAPA)	Cyclotron
^86^Y	14.7 h	31.9% (660.0 keV)	γ (637 keV; 32.6%) γ (1076 keV; 82.5%) γ (1153 keV; 30.5%) γ (1920 keV; 20.8%)	Metallic	Complexation (DOTA, DTPA)	Cyclotron
^76^Br	16.2 h	55.0% (1180 keV)	γ (559 keV; 74.0%) γ (657 keV; 15.9%) γ (1216 keV; 8.8%) γ (1854 keV; 14.7%)	Halogen	Covalent (Direct)	Cyclotron
^89^Zr	78.4 h	22.7% (395.5 keV)	γ (909 keV; 99.0%)	Metallic	Complexation (DFO)	Cyclotron
^124^I	4.18 d	22.7% (820 keV)	γ (602 keV; 62.9%) γ (722 keV; 10.3%) γ (1690 keV; 11.1%)	Halogen	Covalent (Direct/prosthetic)	Cyclotron

Several additional considerations must also be taken to rationalize the choice of a positron emitter. Positron energy range is a key factor for intrinsic resolution of PET modality because it has a direct and significant impact on the positron travel distance before annihilation. As a consequence, a high-energy positron will result in an intrinsic resolution loss. In addition, existence of concomitant γ and/or β^−^ emissions will have a major impact on the staff and/or patient radiation dose. It should be noted, to our knowledge, they are currently no allowed maximal recommended activity limitation per isotopes. Dosimetric studies are on progress and first results obtained with trastuzumab showed an effective dose equivalent to the whole body of 45 and 10.8 mSv, respectively for ^89^Zr and ^64^Cu (17, 18). Of course, these preliminary results are only indicative because, the dosimetric data are also under the dependence of the vector pharmacokinetic profile, As consequences, the dosimetry for smaller mAb derivatives like nanobodies is lower (shorter *in vivo* stay, due to a fast renal clearance). For example, the dosimetry induced by 185 MBq ^68^Ga-2Rs15d nanobody is close to the dosimetry due to the classical 370 MBq ^18^F-FDG injection with, respectively, 4 and 7 mSv (19).

The chemical nature of the isotope presents a direct impact on the immunoprotein radiolabeling (20). Classically in radiopharmaceutical practices, radionuclides may belong to the radiohalogen or radiometal family, Radiohalogens like iodine-131 could be directly radiolabeled to the amino-acid chain on an aromatic residue (such histidine or more usually on a tyrosine) after gentle oxidation of iodide (21). This effective method presents some limits (mAb sensitive to oxidizing environment, low stability of radioactive mAb,…) and indirect radiolabeling could be envisaged. The indirect radiohalogen labeling is based on the use of prosthetic group intermediate (5, 22–25) like Bolton-Hunter reagent, organostanyl compound or iodonium salts (26–29).

Radiometals form very stable coordination complexes with a great variety of ligands, including linear diethylenetriaminepentaacetic acid (DTPA) derivatives, macrocyclic [1,4,7,10-tetraazacyclododecane-1,4,7,10-tetraacetic acid (DOTA) polyaminocarboxylic or desferrioxamine (DFO) derivatives] (30–32). These ligands are transformed in bifunctional chelating agent (BCA) capable of reacting with proteins to form a stable covalent bond with lysine residue, cysteine residue or synthetic bioorthogonal [click-chemistry approach (33)].

Finally, the production way of the isotope has consequences on the cost and in the availability of the radionuclide. The great majority of positron emitters are produced by cyclotron accelerator with a relatively high usual production cost. The half-life of the radionuclide presents a direct impact on the logistic because of decay during the transport. To circumvent this constraint a cyclotron network was built close to nuclear medicine departments for the short half-life isotopes (fluor-18; scandium-44; copper-64). These particular supply chain leads to a particular production schedule without daily availability of all isotopes. To solve this problem, some isotopes like Gallium-68 can be put in generator form (in this case, industrial supplier used germanium-68 with a longer half-life to produce in nuclear medicine department the desired gallium-68 by filiation).

## MAbs and derivatives

The discovery of hybridoma technology by Köhler et al. (34) originally allowed the production of antigen-directed specific mAbs. This discovery paved the way for immuno-PET development but has appeared to be limited by the immunogenicity due to the murine origin of the amino-acid sequence (35). To circumvent this first hurdle, chimeric then humanized mAbs were designed by molecular biology and enabled less immunogenic approach with a better tolerance profile for the patients (36). The murine, chimeric and humanized mAbs are entire (intact) immunoglobulin G (IgG) with a long pharmacokinetic half-life and high weight (150 kDa) which reduces their capacity to diffuse inside the tumor mass.

To adapt these properties (pharmacokinetic and distribution), biochemists and immunochemists have developed some immunoconjugate derivatives exhibit reduced half-lives and improved tumoral penetration. The overall characteristics of these protein structures are summarized on Table 2.

**Table 2:** Usable antibodies and derivatives.

**Name**	**IgG**	**F(ab')_2_**	**F(ab)**	**Minibody**	**ScFv**	**Nanobody**	**Affibody**
Structure	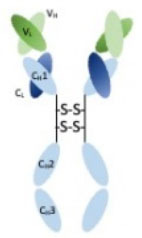	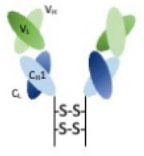	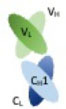	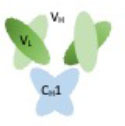	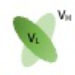	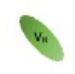	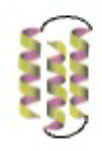
Size	150 kDa	110 kDa	50 kDa	≈ 75 kDa	≈ 25 kDa	15 kDa	14 kDa
Valence	Divalent	Divalent	Monovalent	Divalent	Monovalent	Monovalent	Monovalent
Immunogenicity	Mouse > chimeric > humanized	Low	Low	Low	Low	Low	Low
PK half-life	Few days to several weeks	≈ 24 h	≈ 4 h	Few hours	≈ 1 h	≈ 1 h	< 1 h
Elimination	Liver	Liver	Kidney	Liver	Kidney	Kidney	Kidney

The first improvement was the use of mAbs fragments like F(ab')_2_ and F(ab). These smaller protein fragments consist in the reduction/digestion of initial mAbs by enzymes (pepsin and papain). The F(ab')_2_ and F(ab) fragments are smaller (110 and 50 kDa, respectively) than the native mAbs but preserve the affinity and the specificity for the tumor antigen. F(ab')_2_ fragments of mAbs were radiolabeled with ^64^Cu in preclinical studies in breast cancer (37) or in lymphoma (38) animal models. Results showed a rapid and sustained uptake in tumor with promising and encouraging perspectives for the clinical evaluation of malignant pathologies while delivering a lower total body radiation dose compared to the entire mAbs. F(ab) fragments radiolabeled with ^68^Ga (39) or ^64^Cu (40) showed similar results with a shorter pharmacological half-life. In terms of biodistribution, F(ab) fragments present the characteristic of having a mass weight lower than the renal size cut-off (30–50 kDa) and consequently of being eliminated by the urinary tract (41). As consequence, F(ab) fragments cause high and persistent localization of the radioactivity in the kidney which could be reduced by a metabolizable linker between F(ab) fragment and isotope part of the construct (42).

Building on these successes, immunochemical engineers have turned to small synthetic proteins which contains only the antigen recognition domain of the initial mAb such minibodies, fusion protein-like single chain variable fragments (ScFv) or nanobodies. Minibodies consist of constant heavy chain (CH) and variable light (VL) and variable heavy (VH) domains, ScFv constructs are made of solely VL/VH domains and nanobodies has only the VH domains. ^124^I and ^89^Zr radiolabeled minibodies validated the feasibility proof-of-concept of immuno-PET in prostate cancer animal model (43). In breast cancer animal model, ScFv fragments radiolabeled with ^68^Ga (44) showed *in vivo* promising results. On their sides, nanobodies are subjects of intense preclinical research (45–47). These engineered antibody-based proteins demonstrate their capacity to be used in nuclear medicine field with β^+^ emitters for immuno-PET applications (48, 49). To date, the proof-of-concept was validated at a preclinical level and exploratory work in human is started (50).

More recently, synthetic proteins (three-helix scaffold) generated from a phage display library were produced with a high affinity against an antigenic structure. These proteins, named affibodies, have been prepared against different cancer targets for PET applications and were studied *in vitro* with promising results (51–53).

To optimize the tumor-to-background ratio, recent advances of nuclear medicine demonstrate the great interest of pretargeting approach (54). This technic involves a preliminary injection of a non-radioactive bispecific antibody followed by an injection of a radiolabeled bivalent hapten (peptide or small organic molecule). This system allows to bypass the slow clearance of bispecific mAbs. In this particular case, a bispecific mAb recognizes the tumor antigen with a high specificity by one of its two recognition domains. The second recognition domain is used to bind the radioactive hapten which is injected after the bispecific mAb within an optimal delay. The unbound hapten is rapidly cleared from the blood circulation via the renal system and allows, de facto, an increase of the immuno-PET image contrast. The hapten approach is not the only one feasible for pretargeting and bio-orthogonal reactions based on click-chemistry has recently been considered with successful results in preclinical studies (55, 56). The mAb (entire IgG or derivative) is *in vitro* prefunctionalized with a chemical clickable function. The intra-venous injection of this compound is followed by the injection of a radioactive compound with clickable complementary function and a specific covalent bond between these two components is *in vivo* formed. The relatively short pharmacokinetic half-life of the radioactive clickable moiety allows the use of shorter half-life isotopes and a renewed interest for fluorine-18 in immunoPET applications (57).

## Example of clinical proof-of-concept

### Antigen targeting in solid tumors

One of the first clinical proof of immuno-PET interest has been reported by Divgi et al. (58) with a chimeric mAb (cG250, girentuximab) directed toward the carbonic anhydrase IX (CAIX) cell-surface antigen particularly overexpressed in clear cell renal cell carcinoma (ccRCC). In this phase I clinical study, the mAb was ^124^I-radiolabeled and shows a very good sensitivity and specificity (respectively 94 and 100%) for ccRCC. Consolidated results obtained during the REDECT trial (Divgi JCO 2013) showed more robust results with sensitivity and specificity (respectively 86.2 and 85.9%). As consequences, immuno-PET can accurately and non-invasively assess the initial diagnostic of cancer without the inherent risk of biopsies. Despite this promising results, iodine-124 has physics drawbacks and zirconium-89 has been preferred in many subsequent clinical trials with girentuximab (59, 60). CAIX is also upregulated in various solid tumors and many clinical trials are currently underway for different tumor sites such as urothelial/bladder cancer (61) or triple negative breast cancer (OPALESCENCE Study Clinicaltrials.gov identifier NCT 04758780).

One of the first studied antigen for nuclear medicine purpose is the carcinoembryonic antigen (CEA). Different anti-CEA mAbs have been radiolabeled for therapeutic applications with ^131^I or for SPECT imaging with ^111^In. It is quite natural that it has been used in various immuno-PET studies. For example, AMG211 mAb was a bispecific antibody directed toward CEA and CD3 (62). AMG211 has been radiolabeled with ^89^Zr to evaluate the tumor uptake in relapsed/refractory gastrointestinal adenocarcinoma patients (Clinicaltrials.gov identifier NCT 02760199). M5A mAb is also an anti-CEA mAb and was radiolabeled with ^124^I for the detection of CEA positive colorectal cancer that has spread to the liver (Clinicaltrials.gov identifier NCT 03993327) or with ^64^Cu for the diagnosis of CEA positive rectal cancer (Clinicaltrials.gov identifier NCT 05245786) or in gastrointestinal, lung, medullary thyroid and breast cancers (Clinicaltrials.gov identifier NCT 02293954). CEA can also be targeted by a bispecific mAb for a pretargeting approach. TF2 is an engineered bispecific mAb of 157 kDa formed by the Dock-and-Lock^®^ procedure from the anti-hapten Fab fragment recognizing the histamine-succinyl-glycine (HSG) motif and two humanized anti-CEA fragments. During the procedure (iTEP-CMT; Clinicaltrials.gov identifier NCT 02293954), non-radioactive TF2 mAb was injected in medullary thyroid carcinoma patients and 24 h later, ^68^Ga-di-HSG hapten was injected. The preliminary results demonstrated a promising sensitive and specific imaging method (63, 64) with a requirement in terms of optimization, both for the dosing (TF2 and hapten) and administration schedules (64). Similar and encouraging results were obtained in other CEA overexpressed solid tumors such colon (65) or breast cancer (66) with this pretargeted immuno-PET approach.

Finally, as another example to illustrate the large and recent evolution of immuno-PET capacities, cancer antigen 6 (CA6) can be targeted with a radiolabeled Fab (67). CA6 is a neuraminidase-sensitive and periodic acid-sensitive sialic acid glycoconjugate often overexpressed in various carcinoma such pancreas, ovary, breast and bladder (68). ^64^Cu-anti-CA6-Fab was clinically used in ovarian and breast cancer (Clinicaltrials.gov identifier NCT 02708511).

One of the most widely used mAbs in clinical practice is trastuzumab. Trastuzumab is directed against Human Epidermal growth factor Receptor 2 (HER2) which is particularly overexpressed in breast cancer. In 2010, Dijkers et al. (69) reported a feasibility study of ^89^Zr-trastuzumab in HER2-positive metastatic breast cancer and has shown the immuno-PET capacity to detect most of the known lesions and some that had remained unnoticed with other diagnostic modalities (CT, MRI or bone scans). Based on this first encouraging results, many clinical trials (70, 71) have been conducted to evaluate the tumor uptake of ^89^Zr-trastuzumab in breast tumor and in their metastatic lesions. The results have shown medium sensitivity of 75.8% (57.7–88.9%) and specificity of 61.5% (31.6–86.1%). These scattering of preliminary results seems to be explained by the high liver uptake which is strongly dependent of the non-radiaocative mAb injected. Indeed, when the authors remove the liver metastases from their interpretation, the SUV_max_ appears to be significantly higher in HER2 positive compared to HER2 negative patients. In this study, immuno-PET approach demonstrated its ability to identify the intrapatient heterogeneity of ^89^Zr-trastuzumab uptake in 20% of patient with multiple lesions. This intrapatient heterogeneity could be explained by sampling error during biopsy, heterogeneous intratumoral distribution of HER2 antigen or expression change of HER2 status since the last biopsy (72). Nevertheless, ^89^Zr-trastuzumab has the potential to characterize the whole body HER2 status of all the tumor and metastatic sites thus obviating repeated tissue biopsies to assess the intrapatient heterogeneity. Another advantage of the immuno-PET in these clinical circumstances is that it allows a quantitative evaluation of the target expression to optimize the future therapeutic dose and allows a better evaluation of the *in-vivo* penetration (ability to cross the physiologic barrier) of mAbs in the tumor mass comparatively to the *ex-vivo* glass-slide immunohistochemistry (IHC) provided by cytology (73). A possible future for this dose finding may be clinically translated to predict and to monitor the HER2-targeted therapeutics treatments (74). The tumor uptake of ^89^Zr-trastuzumab is related to the target concentration but there is still a pitfall due to the small range for distinguishing IHC classes 1 to 3 with relatively constant SUV. Currently, the whole-body quantitative imaging objective with HER2 immuno-PET has not been reached and requires a normalization of the non-radioactive trastuzumab mass dose injected to minimize the accumulation in health tissue and to maximize the contrast with cancer lesions (69, 73). Nevertheless, this quantitative approach of the *in vivo* status of HER2 in patients with metastatic breast cancer is proving to be an interesting tool in predictive of respond and benefit for women (ZEPHIR clinical trial) which receiving antibody drug conjugate such trastuzumab emtansine (T-DM1) as second line treatment of HER2+ metastatic breast cancer (75, 76). The ZEPHIR clinical trial categorized patients into 4 subgroups in function of the tracer uptake (^18^F-FDG and ^89^Zr-trastuzumab) patterns and allows a very good prognostic in response evaluation. To optimize the radiation safety and patient radiation dose, copper-64 was used by Mortimer et al. (77–79). ^64^Cu-trastuzumab using in HER2 positive breast cancer patients showed a rapid tumor and metastasis uptake of the radiolabeled mAb in a similar way than ^89^Zr (80). A promising clinical trial (Clinicaltrials.gov identifier NCT 05376878) is currently in progress to determine the ability of ^64^Cu-trastuzumab immuno-PET to detect positive brain metastases of breast cancer. HER2 antigen is also overexpressed in ~20% of esophagogastric adenocarcinoma and ^89^Zr-trastuzumab showed promising results (safe and high-quality images) in patients with HER2 positive tumors (81). Same promising results were obtained in gastric cancer with ^64^Cu-trastuzumab (Clinicaltrials.gov identifier NCT 01939275). In regards of the nanobodies applications, a phase I study confirmed the capacity of ^68^Ga-HER2-nanobody to provide safe and informative uptake in breast carcinoma (82).

Despite the very large number of clinical trials using small molecules, the Prostate-Specific Membrane Antigen (PSMA) can also be targeted by mAbs. PSMA is particularly overexpressed in prostate adenocarcinoma and J591 mAb was firstly radiolabeled by ^124^I then by ^89^Zr (83). The first-in-human use of ^89^Zr-J591 (84) showed a correlation between the uptake of ^89^Zr-J591 and the tumor aggressiveness. These first results were confirmed by a phase I/II clinical trial and demonstrated a superior targeting of bone metastases lesions compared to conventional imaging modalities (85). In prostate cancer, ^64^Cu radiolabeled anti-PSMA engineered humanized mAb (HuX592r) is currently under clinical trial (CUPID Study, Clinicaltrials.gov identifier NCT 04726033) to determine the safety, pharmacokinetic, whole body distribution and radiation dosimetry of this possible new immuno-PET modality.

Encouraging results were observed with the epidermal growth factor receptor (EGFR) where ^89^Zr-cetuximab (86) has provided additional information on advanced head and neck cancer (ARTFORCE study, Clinicaltrials.gov identifier NCT 01504815) for an enlightened choice of the most effective treatment between cisplatin or cetuximab (87, 88).

### Antigen targeting in hematologic cancers

Blood cells and hematopoietic stem cells located in bone marrow are well-known to be radiosensitive. As a consequence, hematological diseases such as B-cell non-Hodgkin lymphoma or multiple myeloma are highly studied malignancies in nuclear medicine. Twenty years ago, overexpressed B-lymphocyte antigen CD20 provided an interesting target for β^−^ radioimmunotherapy (RIT) applications with ^90^Y or ^131^I radiolabeled mAbs. At the same period, ^86^Y has gained interest as an interest surrogate for immuno-PET complement of ^90^Y-ibritumomab-tiuxetan RIT (89). Due to the ^86^Y physical drawbacks, ^89^Zr-ibritumomab-tiuxetan was preferred for immuno-PET clinical trial. Rizvi et al. (90) conducted a prospective clinical study to analyze the biodistribution and the radiation dosimetry of ^89^Zr-ibritumomab-tiuxetan prior and after ^90^Y-ibritumomab-tiuxetan RIT. This study has confirmed the potential value of immuno-PET to predict and to optimize RIT as individualized treatment. CD20 antigen is overexpressed in diffuse large B-cell lymphoma, commercial rituximab mAb has an indication on this pathology and can be functionalized with desferrioxamine derivatives for ^89^Zr radiolabeling (91). ^89^Zr-rituximab clinical trial showed a strong correlation between the tumor uptake and CD20 expression (92) with a promising expectation to predict the benefit of “cold” rituximab therapy and to guide individualized treatment. To date, a clinical trial with ^64^Cu-rituximab (Clinicaltrials.gov identifier NCT 01598558) was scheduled but withdrawn without explications in non-Hodgkin lymphoma and could, in a near future, provide new information for immuno-PET development. Daratumumab is a mAb that targets CD38, an antigen overexpressed in nearly all myeloma cells. ^89^Zr-daratumumab was synthesized and showed in a first-in-human study (Clinicaltrials.gov identifier NCT 03665155) a highly sensitive detection of multiple myeloma. These preliminary results allowed a good bone localization before therapy, the quantification of the disease burden before therapy, the best responders for “cold” daratumumab therapy and a detection of minimal residual disease after therapy (93). Daratumumab was also ^64^Cu radiolabeled by Krishnan *et al*. and has allowed whole body images of the multiple myeloma expansion. This ^64^Cu-daratumumab clinical study (Clinicaltrials.gov identifier NCT 03311828) showed a better concordance (94) between biopsy results and ^64^Cu-daratumumab than classical ^18^F-FDG (uptake due to inflammatory region). A future phase II clinical trial is scheduled to determine the sensitivity and the specificity of ^64^Cu-daratumumab in multiple myeloma.

### Antigen targeting of the tumor microenvironment

Recent advances in oncology have revealed the greatest interest of the tumor microenvironment in the disease proliferation process. Tumor microenvironment is an important site of immunologic response and tumor-infiltrating T-cell can be targeted by an anti-CD8 minibody radiolabeled with ^89^Zr (^89^Zr-DfIABM2C). Phase I clinical trial showed promising results in 15 patients to predict early response to immunotherapy (95). The tumor response is under the regulation of immune check point and recent advances in this immunologic field showed the great importance of PD1/PDL1 system (96). Research on these immune check points has identified the program cell death protein (PD1) and the program cell death ligand (PDL1) to have a key role in this process. Overexpression of PD1/PDL1 axis is associated with a poorly patient prognostic in a large range of cancers (97, 98). The potential interest of non-invasive PET imaging of tumor PD1/PDL1 expression is therefore of prime importance for the patient care (99). One of the most used mAb specifically for PD1 is pembrolizumab which could be radiolabeled with ^89^Zr. ^89^Zr-penbrolizumab remained stable in blood circulation with a classical accumulation in liver and spleen tissues (100). Some clinical trials (Clinicaltrials.gov identifiers NCT 02760225; NCT 03065764) were done (or in progress) with ^89^Zr-penbrolizumab and has shown uptake in tumor lesions correlated with treatment response (101). This study was performed in a small patient cohort (14 patients enrolled) and show promised results but need to be confirmed by a larger clinical trial. Similar results were obtained with ^89^Zr-atezolizumab (directed against PDL1) in lobular breast cancer during the ImaGelato clinical study (Clinicaltrials.gov identifier NCT 04222426) (102) or with ^89^Zr-durvalumab in non-small cell lung cancer (103).

To provide increase oxygen and nutrients supply for the growing tumor, tumor cells induct neoangiogenesis by vascular endothelial growth factor (VEGF) secretion. VEGF receptors located at the surface of the endothelial cells can be targeted by mAbs such bevacizumab. ^89^Zr-labeled bevacizumab was prepared and administered in patients with non-small cell lung cancer and showed a correlation between tumor-uptake and progression-free survival and overall survival after treatment (104).

## Conclusion

Today, the immuno-PET field is rapidly progressing and allows to provide essential information for the care management of each patient. To meet the need of personalized medicine era where each patient is unique, immuno-PET provides repeatable, non-invasive whole-body information of biomarkers mapping. Many preclinical developments and clinical proof-of-concepts have been done or are ongoing. The recent clinical developments of immuno-PET have confirmed the potential of mAbs (and their derivatives) as companion diagnostic to determine, whether or not, a patient will respond to a targeted therapy. Nevertheless, the majority of proof-of-concept clinical studies have been performed on small patient samples and require larger studies to confirm their potential as predictive imaging biomarkers. Currently, the main limitation for these clinical studies with great patient numbers are often limited by the costs and the availability limitation of isotopes.

The original phenotypic information provided by immuno-PET currently allows *in vivo* access of several pieces of information on the tumor aggressivity associated to a patient outcome prognostic. The power of this molecular imaging modality seems to be able to show very informative data on the intra-tumor and intra-patient variability on molecular biomarkers expression and consecutively in phenotypic heterogeneity of the entire disease burden. Moreover, in comparison with “gold-standard” biopsies, immuno-PET allows to acquire *in-vivo* knowledge of the intra-tumoral penetration/biodistribution of the mAb without inherent-risk of tumor seeding during needle biopsy (105).

Beyond the image information, immuno-PET is attractive to study the in-vivo behavior of antibody-based therapies and for understanding their therapeutic efficacities. Within the scope of theranostic approach, the use of β^+^/β^−^ pair (respectively for diagnostic then therapy) radiolabeling the same mAb is very promising because the same distribution/pharmacokinetic is expected. Immuno-PET allows in theranostic the determination of the patient dosimetry to optimize the cumulated activity and to predict the therapy response by a quantitative measurement of the tumor-antibody uptake. Molecular imaging by immuno-PET with mAbs or their derivatives will play a pivotal role in the close oncologic future to tailor a customized therapy for each patient.

## Author contributions

CB, CR, FK-B, and CB-M are nuclear medicine physicians. They have worked for many years on the development of immuno-PET approach in oncology. AL is an endocrinologist with an expertise in immuno-PET approach for neuroendocrine tumors. MC is pharmacist, head of the nuclear oncology research team, with a broad expertise in immunology. MB is radiopharmacist with a long term wide experience of radiopharmaceutical development, mainly for immuno-PET applications. All authors contributed to the article and approved the submitted version.

## Conflict of interest

The authors declare that the research was conducted in the absence of any commercial or financial relationships that could be construed as a potential conflict of interest.

## Publisher's note

All claims expressed in this article are solely those of the authors and do not necessarily represent those of their affiliated organizations, or those of the publisher, the editors and the reviewers. Any product that may be evaluated in this article, or claim that may be made by its manufacturer, is not guaranteed or endorsed by the publisher.
